# The effect of the social support on PTSD and PTG about university student volunteers in the prevention and controlling of coronavirus: with coping style as the intermediary

**DOI:** 10.3389/fpsyg.2023.1152823

**Published:** 2023-05-22

**Authors:** Ranran Hao, Peiyu Han, Liangsheng Wang, Yong Zhang

**Affiliations:** School of Education and Psychology, Southwest Minzu University, Chengdu, China

**Keywords:** epidemic prevention, university student, volunteers, post-traumatic stress disorder, posttraumatic growth, social support, coping style

## Abstract

To investigate the relationship among post-traumatic stress disorder (PTSD), posttraumatic growth (PTG), social support, and coping style of university student volunteers in the prevention and control of the coronavirus in 2020, a total of 2,990 university student volunteers (students who are enrolled in a university and involved in volunteer activities) from 20 universities in Sichuan Province participated in the prevention and control of the epidemic were investigated when March 20–31, 2020 when the coronavirus first occurred using the post-traumatic stress disorder questionnaire, posttraumatic growth questionnaire, university student social support questionnaire and coping style questionnaire. The results showed that (1) 7.06% of university student volunteers had some degree of PTSD symptoms (the total PCL-C score was 38–49), and 2.88% had obvious PTSD symptoms, (2) PTSD level of university student volunteers was significantly positively correlated with negative coping style, and significantly negatively correlated with social support and positive coping style; on the contrary, the PTG level is significantly positively correlated with social support and positive coping styles, and (3) Positive coping style plays a partial mediating role in the influence of social support on PTG; in the influence of social support on PTSD, the mediating effect of positive or negative coping style was not significant. These results show that in the prevention and control of the coronavirus, the positive coping style and social support of university student volunteers can positively predict the PTG level of them, while the negative coping style can positively predict the severity of their PTSD symptoms. Among them, a positive coping style plays a partial mediating role in the influence of social support on the PTG level.

## 1. Introduction

In 2020, the coronavirus disease (COVID-19) pandemic swept the world with high speed, wide range, and multi-channel transmission, which is highly dangerous to people’s health and has a ripple effect on all aspects of human life as we know it (Nicola et al., 2020), making it a public health emergency of international concern. The study of “coronavirus” has become a hot research topic in academia (Chen et al., 2020; Li and Shan, 2020). In response to the epidemic situation, which is long-lasting, widespread and dangerous, our people, under the unified leadership of the Party and the State, concentrated human, material and financial resources to support the serious areas of the epidemic at the first time, and carried out strict preventive and control measures to control the epidemic situation. Among them, many university student volunteers have joined the epidemic prevention and control operation, carrying out vaccination personnel screening, household publicity, temperature measurement at the entrance of public places, “three codes” checking and other work. They played an important role in making up for the lack of rescue force, relieving the government’s pressure to fight the epidemic and meeting the needs of the society (Li, 2020). It is inevitable that the university student volunteers who go to the front line of epidemic control will experience more serious trauma exposure. Therefore, we believe that university student volunteers in the outbreak prevention and control have a higher degree of trauma exposure than the general public, and under the premise that trauma exposure is considered a prerequisite or primary factor for the occurrence of PTSD (Dai et al., 2014), we believe that the mental health of university student volunteers in the outbreak prevention and control needs more attention.

PTSD or posttraumatic stress disorder, is a posttraumatic pathological response that manifests as intrusive symptoms, avoidance symptoms, heightened alertness symptoms, negative cognitions, and mood changes (Wang and Jiang, 2002). According to the psychological model of risk (Svenson, 1988), strong physical and psychological reactions such as panic, anxiety, and other adverse psychological reactions may occur when individuals are exposed to more than acceptable levels of risk. In the prevention and control of the COVID-19 pandemic, the volunteer group may witness the painful state of the infected person, constantly worry that they will be infected when they are in close contact with the infected person, and be under the anxiety and fear of the high-risk environment for a long time, which provides multiple risk factors for PTSD generation. In addition, the use of social isolation/lockdown as a strategy to contain the spread of the virus showed more severe trauma and depressive symptoms in students who had previous psychological and psychiatric contact with mental health services. A problematic mindset of “all or nothing” was the strongest predictor of eventual traumatic distress and was primarily associated with psychological distress, anxiety, depression, and post-traumatic symptoms (Giusti et al., 2020). Some studies have pointed out that there are three main characteristics of PTSD in university students: delayed in time, superimposed in events and psychologically curable (Wang and Qiao, 2019). The manifestation of PTSD symptoms in university student volunteers at work may emerge and be superimposed in degree and impactfulness at the tail stage or after the end of prevention and control work. Also based on its psychologically curable nature, the trauma status of university student volunteers needs more attention.

With the development of trauma psychology, PTG, which has received attention in contrast to PTSD, has continued to become a recent research hotspot for researchers. Posttraumatic growth (PTG) refers to the positive changes in psychological aspects experienced by individuals in the process of struggling with negative life events and situations that are traumatic, not caused by the traumatic event itself, but through the individual’s struggle with the traumatic event (Tedeschi and Calhoun, 1995). The typical experience of PTG is a greater appreciation for life, a stronger sense of spiritual connection or faith, recognition of new opportunities, acknowledgement of personal power, and/or a stronger sense of connection with others (Dominick, 2022). It has been shown that symptoms of moderate depression are predictive of PTG in a sample of university students and that moderate depressive conditions and associated distress can promote motivation to overcome the psychological consequences of traumatic events (Bianchini et al., 2017). A recent study points to the importance of how university students in the pandemic area cope when faced with problems caused by COVID-19 in themselves or those around them, adjusting their psychological stress response, and gaining experience and growth in the fight against the pandemic. Researchers constructed a mediated regulation model to examine the effects of invasive rumination on university students’ creativity during the COVID-19 pandemic, as well as the mediating role of post-traumatic growth and the moderating role of psychological resilience. The results showed that invasive rumination directly influenced creativity and also indirectly influenced creativity through post-traumatic growth. Meanwhile, the correlation between invasive rumination and post-traumatic growth was stronger when the level of psychological resilience was higher (Xu et al., 2022).

So is there some relationship between PTG and PTSD? In this regard, a researcher studied the relationship between PTG and PTSD in elementary school students at 6, 12, and 18 months after the earthquake and found that the relationship differed at different time periods. The two showed a mutually predictive relationship in different directions at different time periods, and contextual changes also affected the relationship. It is also worth noting that this study also explored the relationship between social support and PTSD and PTG, and found that social support was predictive of PTSD and PTG at different time periods (Zhou et al., 2017).

According to the post-traumatic growth model proposed by Tedeschi and Calhoun (1996), social support and coping styles are important factors influencing the development of PTG. In Schaefer and Moos’ (1998) crisis-growth model, it is also suggested that a supportive environment not only provides the necessary resources for recovery after trauma but also creates a more reassuring and secure atmosphere. Social support is a key protective factor in the psychological adjustment of individuals to traumatic events (Mitchell et al., 2022). Most psychiatric disorders may arise from the dynamic interaction between neuroscience and social science (Maj, 2014). Therefore, it is important to see the importance of personal and social factors that can positively modulate the response to traumatic events and move a person’s psyche in a healthier direction. Good social support has a positive effect on post-traumatic mind changes in terms of improved cognitive understanding. Therefore, social support may be very important to protect a person from mental health problems, especially in a crisis, such as in a prolonged COVID-19 pandemic (Cao et al., 2022).

According to Bandura’s (2001) reciprocal determinism, environmental determinism requires the involvement of internal human factors to play a role, so when exploring the influence of external factors, the influence of internal human-related activity factors is equally important. In Tedeschi and Calhoun (1996) posttraumatic growth model, coping is the individual’s internal system of coping with the environment. Differences in individual coping styles may be responsible for differences in individual performance. Coping styles as intra-individual factors may constrain the effect of social support on PTSD and PTG. Related studies have shown that the effect of social support on PTG under group traumatic events such as earthquakes is mediated by intra-individual factors such as self-efficacy and self-esteem (Zhou et al., 2019). It also supports the need to consider intra-individual factors when studying the effects of social support on PTG or PTSD. In a mass traumatic event such as the COVID-19 pandemic, the traumatic event is likely to challenge people’s otherwise relatively stable conceptions of self in various ways, and this challenge makes people more likely to feel helpless and powerless, which in turn induces a denial of one’s ability or even worth, and leads to a perception of self as incompetent and worthless (Janoff-Bulman, 2010). These perceptions in turn also influence people’s behavior in response to traumatic events and ultimately the overall mind–body response (Foa et al., 1999). It is evident that traumatic events challenge people intrinsically through an internal process and that the way in which people cope after being challenged cognitively plays an important role in this internal process. The social conditions in which individuals are placed and the coping styles they use may have different effects on PTSD and PTG. However, it is worth noting that the above model addresses the theoretical significance of each factor on PTG and PTSD, but there is still a lack of empirical research support for the public health emergency context, which provides a possible perspective for this study.

Based on this, this study proposes the following hypothesis: there is a mediating role of coping style in the role of social support on PTSD and PTG.

In conclusion, among the factors influencing PTSD and PTG in previous studies, the social support and coping style in a specific situation become factors influencing the difference between individuals in a specific situation. The crisis situations experienced by university student volunteers in this serious epidemic control operation need more attention and research. Based on this, this study intends to investigate the role of social support on PTSD and PTG, and the role and influence of coping style on social support in the group trauma situation under the epidemic, using university student volunteers who participated in the epidemic prevention and control operation as subjects.

## 2. Materials and methods

### 2.1. Participants

In this study, university student volunteers involved in epidemic prevention and control in 20 universities and universities in Sichuan Province were used as the research subjects, and an online survey was conducted from March 20–31, 2020, using the Questionnaire Star program, forwarded to university student volunteers through each university teacher organization. The content included general demographic information, post-traumatic stress disorder status, posttraumatic growth status, coping style and social support and other related information. A total of 3,616 questionnaires were collected, and questionnaires that were completed in too short a time, had too many missing answers, or had the same checkboxes or regularity throughout the questionnaire were excluded. A total of 2,990 valid questionnaires were included in the study, with an effective questionnaire return rate 82.69%; 1,472 were from males and 1,518 were from females; 943 were from science, 1,447 were from arts, and 600 were from engineering; 775 were from freshmen, 684 were from sophomores, 650 were from juniors, 357 were from seniors, and 524 were from postgraduates.

### 2.2. Instruments

#### 2.2.1. PTSD checklist-civilian version

The PTSD Checklist-Civilian Version (PCL-C) was used. This scale was developed according to the 4th edition of the Diagnostic and Statistical Manual of Mental Disorders of the American Center for Posttraumatic Stress Disorder Research to assess the experience of individuals after experiencing psychological trauma under non-war conditions, and the items measured included three dimensions: avoidance/numbness, hypervigilance, and re-experiencing. There are 17 items in the scale, which can be divided into subjective ratings of the traumatic event, repeated reoccurrence experience, avoidance symptoms, heightened alertness and impaired social functioning, and each item is rated on a scale of 1–5, from no effect to very severe effect, according to the psychological feelings after the traumatic event. The cumulative score of the 17 items is the total PCL-C score, and the higher the score, the more severe the stress disorder condition. If the total score was 17–37, there were no significant PTSD symptoms; if the total score was 38–49, there was some degree of PTSD symptoms; if the total score was 50–85, there were significant PTSD symptoms. The Cronbach’s alpha coefficient for the scale in this study was 0.936.

#### 2.2.2. Posttraumatic growth inventory

This study used the Chinese version of the Posttraumatic Growth Inventory (C-PTGI) revised by Geng et al. (2011) to the Posttraumatic Growth Inventory (PTGI) developed by Tedeschi and Calhoun. The scale includes a total of 21 entries in 5 dimensions, namely 7 entries assessing the interpersonal relationship dimension, 5 entries assessing the new possibilities dimension, 4 entries assessing the personal strength dimension, 2 entries assessing the spiritual change dimension, and 3 entries assessing the appreciation of life dimension. The “never” option scored 0 points, the “rarely” option scored 1 point, the “little” option scored 2 points, the “medium” option scored 3 points, the “large” option scored 4 points, and the “maximum” option scored 5 points. “The higher the score, the higher the PTG level. In this study, the Cronbach’s alpha coefficient for the scale was 0.944.

#### 2.2.3. Simplified coping style questionnaire

A simplified coping style questionnaire prepared by Yaning Xie was used, and the questionnaire used a total of 20 entries, and the entries were scored on a 4-point scale. The questionnaire was divided into two subscales: positive coping style and negative coping style. The positive coping style subscale consists of questions 1–12, focusing on the characteristics of positive coping style when individuals encounter stress. Specific behaviors such as “Talking to others about inner troubles,” “Seeking possible advice from others,” “Trying to think and observe the good side of things,” etc. The negative coping style subscale consists of 13–20 questions, focusing on the characteristics of individuals’ negative coping style when they encounter stress. Specific behaviors such as “avoidance of problems,” “meaningless passive waiting,” and “habit of relying on others” were identified. In this study, the Cronbach’s alpha coefficient for the positive coping subscale was 0.898; the Cronbach’s alpha coefficient for the negative coping subscale was 0.798.

#### 2.2.4. Social support scale for university students

The social support scale for university students developed by Yeh and Dai (2008) based on Shuiyuan Xiao’s three-factor model of social support was used. The scale consists of 17 entries divided into three dimensions: subjective support, objective support, and support utilization, and each entry is scored using a 5-point scale. This scale has good reliability and validity, with higher scale scores indicating better social support. The Cronbach’s alpha coefficient of the scale in this study was 0.957.

### 2.3. Data analysis

Data processing in this study was performed using SPSS 23.0 with which Process 2.16 program for statistical analysis of data and Bootstrap mediated effects test. First, descriptive statistics of PTSD status of the university volunteers were conducted and ANOVA was performed to study the differences between volunteers with each PTSD level in each dimension. Secondly, the mean and standard deviation of each factor of PTSD, PTG, social support and coping style were described and Pearson correlation analysis was performed. Finally, mediating effects analysis was performed using the Process procedure.

### 2.4. Common method bias test

Since the instruments used in this study were all self-assessment scales, the data obtained from the measurements may cause covariation, which in turn may lead to systematic errors. Based on this, this study used Harman’s one-way test for statistical control of common method bias. It was found that 11 factors were obtained for both rotated and unrotated, and the first factor variances of the results after rotated and unrotated were 22.1% and 13.75%, respectively, which were less than the critical value of 40%. Therefore, we concluded that there was no significant common method bias in this study.

## 3. Results

### 3.1. Survey of PTSD symptoms in university student volunteers for epidemic prevention and control

Data analysis showed that among the university student volunteers in epidemic prevention and control, 2,693 (90% of the overall number) had no obvious PTSD symptoms, 211 (7.06% of the overall number) had some degree of PTSD symptoms, and 86 (2.88% of the overall number) had obvious PTSD symptoms. To examine the differences between different groups of PTSD symptoms in other factor dimensions, tests for differences in different factors such as PTG, social support and coping styles were conducted, and the results are shown in Table 1.

**Table 1:** Means and standard deviations of coping styles, PTG, and social support of university students under different levels of PTSD in university volunteers (*M* ± SD).

Variables	No significant PTSD symptoms	Have some degree of PTSD symptoms	Significant PTSD symptoms	*F*	*P*
Positive coping	23.03 ± 6.13	21.78 ± 5.47	21.77 ± 6.33	5.60^**^	0.004
Negative coping	8.18 ± 4.16	10.44 ± 3.96	12.64 ± 5.09	72.77^***^	0.000
Subjective support	20.78 ± 3.67	18.24 ± 3.81	17.03 ± 4.19	85.04^***^	0.000
Objective support	26.38 ± 3.87	23.59 ± 4.59	21.03 ± 5.16	118.74^***^	0.000
Support utilization	24.15 ± 4.87	21.68 ± 4.84	19.94 ± 5.02	53.82^***^	0.000
Total social support score	71.32 ± 11.25	63.50 ± 12.20	58.01 ± 13.65	98.156^***^	0.000

*p < 0.05, **p < 0.01, ***p < 0.001.

As can be seen from Table 1, the differences in positive coping styles and negative coping styles among university volunteers with different PTSD symptoms were significant. Among them, positive coping scores were highest for those without significant PTSD symptoms, and negative coping scores were highest for those with significant PTSD symptoms. In the social support dimension, university student volunteers with different PTSD symptoms had significant differences in the total social support score and each dimension. And in all three dimensions, the highest scores were for those without obvious PTSD symptoms and the lowest scores were for those with obvious PTSD symptoms.

### 3.2. Correlation analysis of PTSD with PTG, coping style, and social support

Pearson correlation analysis was conducted on PTSD, PTG, positive and negative coping styles, and social support of university students, and the results are shown in Table 2. In this epidemic prevention and control, the PTSD level of university student volunteers showed a significant positive correlation with negative coping styles, and a significant negative correlation with social support and positive coping styles; PTG level showed a significant positive correlation with social support, positive coping styles, and negative coping styles all showed significant positive correlations. Among them, the correlation between PTG and PTSD in this context was significantly negative.

**Table 2:** Correlation analysis table of social support, coping style, PTSD, and PTG.

	Variables	*M* ± SD	1	2	3	4	5
1	Total social support score	70.38 ± 11.76	1				
2	Positive coping style	22.90 ± 6.10	0.376^***^	1			
3	Negative coping style	8.47 ± 4.28	0.024	0.270^***^	1		
4	PTG	61.51 ± 16.48	0.257^***^	0.460^***^	0.096^***^	1	
5	PTSD	26.86 ± 8.62	−0.369^***^	−1.17^***^	0.261^***^	−0.050^**^	1

*p < 0.05, **p < 0.01, ***p < 0.001.

### 3.3. The mediating role of coping styles between social support and PTG

After normalizing all variables, mediated effects analysis was conducted using the SPSS macro program PROCESS Model 4 under control for grade, gender, and major. A bias-corrected percentile Bootstrap method was used for mediated significance testing, with confidence intervals set at 95%, and 5,000 replicate sampling was performed. The mediating effect of social support on PTSD impact in either positive or negative coping style was not significant (BootLLCI = −0.0051, BootULCI = 0.0165; BootLLCI = −0.0043, BootULCI = 0.0119); the mediating effect of social support on PTG impact in negative coping style was not significant (BootLLCI = −0.003, BootULCI = 0.0083). The mediating effect of social support on positive coping style in the effect of PTG was significant, and the results are shown in Table 3.

**Table 3:** Test of mediating effect of positive coping style in the effect of social support on PTG.

Regression equation (*N* = 2,990)		Fitting index		Coefficient Significance	
Outcome variable	Predictor variable	*R^2^*	*F*	*β*	*t*
Positive coping style		0.15	127.20***		
	Gender			0.53	2.52*
	Major			0.26	1.79
	Grade			0.26	2.62*
	Social support for university students			0.19	21.67***
PTG level		0.22	171.65***		
	Gender			−0.27	−0.50
	Major			0.75	1.99
	Grade			−0.45	−28*
	Positive coping style			1.15	24.36***
	Social support for university students			0.14	5.81***

*p < 0.05, **p < 0.01, ***p < 0.001.

As seen in Table 3, positive coping style positively predicted PTG level (*β* = 1.15, *p* < 0.001), and social support of university students positively predicted PTG level (*β* = 0.14, *p* < 0.001).

The results in Table 4 show that the total effect of social support for university students affecting PTG levels was 0.3631, SE was 0.0250, *p* < 0.001; the direct effect value was 0.1426, SE was 0.0245, *p* < 0.001, the lower limit of BootCI was 0.0944, and the upper limit of BootCI was 0.1907; the indirect effect value was 0.2205, SE was 0.0176, *p* < 0.001, the lower limit of BootCI was 0.1872, and the upper limit of BootCI was 0.2570. The relative effect value of the direct effect was 39.27% and the relative effect value of the indirect effect was 60.73%. Since the lower and upper limits of the BootCI for the indirect effect are greater than 0, it indicates that there is a significant mediating effect of positive coping style in the effect of social support of university students on PTG levels. Since its direct effect is equally significant, it indicates that the mediating effect is partially mediated. The product of its direct effect value and indirect effect value was greater than 0, indicating the existence of other mediators that positively predicted PTG levels. However, the mediating role of negative coping style was not significant in the effect of social support on PTSD levels.

**Table 4:** Indirect versus direct effects of positive coping style in the test of mediating effects in the effect of social support on PTG levels.

	Effect value	Boot standard error	Bootcl lower limit	Bootcl upper limit	Relative effect value (%)
Total effect	0.3631	0.0250	0.3141	0.4121	
Direct effect	0.1426	0.0245	0.0944	0.1907	39.27
Indirect effect	0.2205	0.0176	0.1872	0.2570	60.73

## 4. Discussion

In this study, we found that the PTSD detection rate in the outbreak prevention and control was 9.94% in the university student volunteer population, including all participants with PTSD symptoms. This is much lower than the PTSD detection rate (54.6%) among university student volunteers after the Wenchuan earthquake by Lv (2010), which may be related to the traumatic event itself. Most of the panic about the sudden arrival of the COVID-19 pandemic comes from a sense of unfamiliarity and harm, and some studies have shown that people’s psychological stress increases significantly in the first few months after the COVID-19 outbreak, but after a few months have passed, levels of anxiety, distress and depression begin to decline (Aknin et al., 2021). With the rapid research and analysis by scientists, the characteristics of the new coronavirus are no longer mysterious, while the clinical efficacy of the developed symptomatic drugs has been highlighted, coupled with the efficient prevention and control of the epidemic by the Party and the government, people’s knowledge and confidence in the prevention and control measures of the epidemic are also increasing. Second, although university student volunteers are in the positions most likely to be exposed to the virus, the degree of trauma exposure varies among volunteers, and the degree of trauma exposure in this epidemic prevention and control is different from that of the volunteers in the earthquake zone in Wenchuan. Compared to the more hidden unknown and long lasting trauma exposure in the current epidemic, the more visual and intense stimulation in the earthquake may also have contributed to the high PTSD detection rate among university student volunteers at that time. Although the detection rate of PTSD in the university volunteer population in this epidemic prevention and control is lower than that in other groups of traumatic events, it is noteworthy that there are different degrees of lifetime prevalence of PTSD in different traumatic events (Wang and Jiang, 2002), which poses a great challenge to the mental health of the university volunteer population under the epidemic prevention and control. Currently, two main clinical approaches are used to treat PTSD, namely pharmacotherapy and non-pharmacotherapy. Among them, psychotherapy has a definite effect. Prolonged exposure therapy (PE) and cognitive processing therapy (CPT) are both cognitive-behavioral therapies, and both give full attention to the traumatized population, the former aiming to eliminate the fear by imagining the trauma exposure, and the latter aiming to bring the therapeutic effect into full play by re-establishing the cognitive dysfunction caused by PTSD (Chen et al., 2018). We should pay enough attention to the volunteers involved in the epidemic prevention and control work, and recognize the mental health prevention and control of the volunteer population while controlling the development of the epidemic in a timely manner.

This study also analyzed differences in social support dimensions between university student volunteers with different levels of PTSD symptoms. It was found that there were significant differences in multiple dimensions of social support dimensions, such as subjective support, objective support, and support utilization. The mean scores of the three dimensions showed decreasing results from university volunteers with no PTSD symptoms, to university volunteers with some PTSD symptoms, to university volunteers with significant PTSD symptoms. This study also confirmed the result that social support was significantly negatively correlated with PTSD (*r* = −0.369, *p* < 0.001). According to Xiao (1994) social support theory, subjective, experiential or emotional support, i.e., university volunteers’ more emotional experience and satisfaction of being respected, supported, and understood in the society, affects the production of PTSD. The objective, visible, practical or material direct help that supports university volunteers is itself helping university volunteers to suppress the effects of trauma on the one hand; on the other hand, it also becomes the basis of subjective support. All of the above mentioned subjective and objective supports are related to support utilization. The ability to perceive positive internal experiences, to adopt positive coping styles for oneself, and to use positive coping styles in socially supportive contexts directly affects support utilization. It can be seen that social support utilization is linked to the coping styles of university volunteers.

The present study showed that social support had a significant positive predictive effect on PTG levels, which is consistent with the results of other researchers (An et al., 2018; Zhou et al., 2019). The current study also confirmed the positive predictive effect of two factors, social support and coping style, on the PTG level of college volunteers under epidemic prevention and control in the posttraumatic growth model proposed by Tedeschi and Calhoun (1996). And PTSD in this context showed a negative correlation with PTG, that is, the deeper the university volunteers developed PTSD, the lower their level of post-traumatic growth and the lower their possibility of obtaining healing. Also because, the characteristic that PTSD and PTG can co-exist, it also means that the higher the post-traumatic growth can reduce the PTSD symptoms. According to the two-sided glue model of PTG (Maercker and Zoellner, 2004) PTG includes both positive constructs and self-deception factors. Based on this, university volunteers with different psychological qualities react differently when facing traumatic events. Those volunteers who promptly regurgitated and reconstructed their self-perceptions and self-referred and reassured, although they were in the traumatic environment of the epidemic, their prompt internal reconstruction results suppressed the effect of PTSD triggering factors. The same implies that university student volunteers who fail to respond to the effects of PTSD triggering factors in a timely manner are not only at increased risk of developing PTSD symptoms themselves, but are also less likely to experience regurgitation and reevaluation of their self-perceptions, cueing and reassurance of self, and individuals are more likely to experience intrusive traumatic memories in their cognitive world related to the epidemic prevention and control process (Halligan et al., 2003) and the resulting intrusions make it more difficult for volunteers who are already in a traumatic situation to make positive cognitive evaluations of the traumatic event in a short period of time (Williams and Moulds, 2010; Zhou et al., 2015). Their native power to produce PTG is inhibited.

The present study also confirms the partially mediating role of positive coping style in the effect of social support on PTG. That is, during the effect of social support on PTG levels, part of the role is indirectly realized through other pathways: social support → positive coping style → PTG. Good social support creates a safe and stable atmosphere for university volunteers, which facilitates volunteers to use more positive coping methods to deal with traumatic events. Positive coping styles such as actively confiding in others and more pro-social behaviors further contributed to the cognitive reconstruction and self-soothing of the volunteers after the traumatic experience, and also further enhanced the support utilization of the university volunteers, enabling them to better accomplish their epidemic prevention and control tasks while maintaining their physical and mental health. However, we also found that although negative coping styles had a significant positive predictive effect on PTG, “avoiding problems,” “trying to forget,” and “self-deception” increased university volunteers’ self-soothing and deception. The volunteers’ self-soothing and deception also contributed to PTG to some extent. However, it did not play a mediating role in the path of social support, probably because its lower correlation affected the indirect effect of negative coping styles in the path.

The findings of this study help people ensure healthy lifestyles, promote the well-being of people of all ages, and contribute to the global goals of the 2030 Agenda for Sustainable Development.

## 5. Limitations and future research

The present study also has some limitations. First, in terms of study design, this study did not test trauma exposure separately, and the level of trauma exposure of different university volunteers was not well controlled. Secondly, the study did not use the latest scale based on DSM-5 to measure PTSD, and the choice of the study instrument should be improved. Again, the data collection in this study was done by a convenience sampling method, and the online survey was distributed to students by teachers in Sichuan universities, which did not achieve a good random sampling. Based on the limitations of the epidemic form in Sichuan and the student structure of universities in Sichuan province, no centralized survey was conducted on the epidemic prevention and control work in high-risk areas of the epidemic, and the representativeness of the subject group needs to be improved. Finally, some university volunteers showed obvious clinical symptoms of PTSD, but this study did not use clinical criteria for further study. Future studies could further consider the relationship with other variables (illness, having a family member/friend who is ill/died, etc.) as well as controls for the length of time volunteers spend volunteering. There were studies using volunteer students and graduates who were experts in the field of civil defense and who had been adequately trained in their professional culture, enabling them to deal flexibly with a variety of emergencies. The results showed that during the COVID-19 pandemic, more than 90% of volunteers demonstrated good mental health and extensive use of functional coping strategies, and less experienced volunteers showed better emotional profiles compared to their colleagues with 10 or more years of experience. In summary, those who work in the volunteer sector will demonstrate a positive, strong sense of helpfulness and an awareness of their group identity, enabling all members to experience a sense of well-being, belonging, solidarity and cohesion (Roncone et al., 2021). In fact, recent studies have also shown that volunteers who served during the COVID-19 pandemic felt “happy” and satisfied despite the initial distressing conditions they experienced. Their ability to experience happiness alongside the inevitable stress helped them to reduce the psychological stress of doing this important but demanding work (Mo et al., 2022).

## Data availability statement

The original contributions presented in the study are included in the article/supplementary material, further inquiries can be directed to the corresponding author.

## Ethics statement

Ethical review and approval was not required for the study on human participants in accordance with the local legislation and institutional requirements. The patients/participants provided their written informed consent to participate in this study.

## Author contributions

PH and RH contributed to conception and design of the study. LW performed the statistical analysis. RH wrote the first draft of the manuscript. PH and YZ contributed to manuscript revision. All authors contributed to the article and approved the submitted version.

## Funding

Special fund project for basic scientific research business expenses of central universities (2016NGJPY08), National Natural Science Foundation of China Youth Program (81303087).

## Conflict of interest

The authors declare that the research was conducted in the absence of any commercial or financial relationships that could be construed as a potential conflict of interest.

## Publisher’s note

All claims expressed in this article are solely those of the authors and do not necessarily represent those of their affiliated organizations, or those of the publisher, the editors and the reviewers. Any product that may be evaluated in this article, or claim that may be made by its manufacturer, is not guaranteed or endorsed by the publisher.
